# Spatial Changes in Microbial Communities along Different Functional Zones of a Free-Water Surface Wetland

**DOI:** 10.3390/microorganisms8101604

**Published:** 2020-10-18

**Authors:** Mikhail V. Semenov, George S. Krasnov, Ksenia Y. Rybka, Sergey L. Kharitonov, Yulia A. Zavgorodnyaya, Anna V. Yudina, Nataliya M. Shchegolkova

**Affiliations:** 1 Department of Soil Biology and Biochemistry, Dokuchaev Soil Science Institute, 119017 Moscow, Russia; anna.v.yudina@gmail.com; 2 Laboratory of Postgenomic Research, Engelhardt Institute of Molecular Biology Russian Academy of Sciences, 119991 Moscow, Russia; gskrasnov@mail.ru; 3 Institute of Water Problems RAS, Gubkin Street, 3, 119333 Moscow, Russia; kseniarybka@gmail.com (K.Y.R.); nshegolkova@gmail.com (N.M.S.); 4 Faculty of Soil Science, Lomonosov Moscow State University, 119991, Leninsky Gory, GSP-2, 119234 Moscow, Russia; sergey050894@gmail.com (S.L.K.); zyu99@mail.ru (Y.A.Z.)

**Keywords:** constructed wetland, microbiome, 16S rRNA gene amplicon sequencing, organic pollutants, particle size distribution, wastewater treatment, storm water

## Abstract

Constructed wetlands (CWs) are complicated ecosystems that include vegetation, sediments, and the associated microbiome mediating numerous processes in wastewater treatment. CWs have various functional zones where contrasting biochemical processes occur. Since these zones are characterized by different particle-size composition, physicochemical conditions, and vegetation, one can expect the presence of distinct microbiomes across different CW zones. Here, we investigated spatial changes in microbiomes along different functional zones of a free-water surface wetland located in Moscow, Russia. The microbiome structure was analyzed using Illumina MiSeq amplicon sequencing. We also determined particle diameter and surface area of sediments, as well as chemical composition of organic pollutants in different CW zones. Specific organic particle aggregates similar to activated sludge flocs were identified in the sediments. The highest accumulation of hydrocarbons was found in the zones with predominant sedimentation of fine fractions. Phytofilters had the highest rate of organic pollutants decomposition and predominance of *Smithella*, *Ignavibacterium,* and *Methanothrix*. The sedimentation tank had lower microbial diversity, and higher relative abundances of *Parcubacteria*, *Proteiniclasticum*, and *Macellibacteroides*, as well as higher predicted abundances of genes related to methanogenesis and methanotrophy. Thus, spatial changes in microbiomes of constructed wetlands can be associated with different types of wastewater treatment processes.

## 1. Introduction

Water pollution is a challenging problem that affects water resources, ecosystems, biodiversity, and human health. Nowadays, conventional wastewater technologies are becoming insufficient, due to more and more stringent emission standards for pollutants. Moreover, they are expensive and not feasible for widespread application in rural areas [1,2]. Therefore, environmental technologies have received more attention and development as alternative solutions for wastewater treatment in recent years [3].

Constructed wetlands (CWs) have become widely used as a cost-effective and useful alternative to conventional technologies for wastewater treatment [3,4,5,6,7]. They were designed to utilize the same processes that occur in natural wetlands but perform them within a more controlled environment [6,8]. CWs could be applied to all types of wastewater including sewage, industrial and agricultural wastewaters, or landfill leachate [7,8,9,10,11]. CWs have been successfully used to remove a wide range of pollutants from wastewater such as organic compounds, suspended solids, pathogens, and nutrients [12,13,14,15,16,17,18], as well as pharmaceutical and personal care products [19,20,21]. Compared to conventional treatment systems, treatment in CW is cheaper, as well as easier to operate and maintain [3]. In addition, CWs could be modified to provide other ecosystem services such as flood control, carbon sequestration, or wildlife habitat [6,22]. The main difference between CW and systems with activated sludge or biofilters is a long hydraulic retention time (at least three days), which results in a specific microbial community capable of decomposing complex organic pollutants.

Environmentally, CWs may be considered as complicated ecosystems that involve vegetation, sediments, and with associated microbial communities that are all constantly exposed to incoming wastewater [6,23,24]. The microbial community in a CW consists of autochthonous (indigenous) and allochthonous (exogenous) microorganisms [25,26]. Autochthonous microorganisms are able to survive and grow in wetland systems during purification processes, while most allochthonous microbes (including pathogens entering with wastewater) do not survive or have any functional importance in the wetland environment [25,27,28]. Autochthonous microorganisms mediate many processes involved in the purification of wastewater in CWs. These processes include mineralization of organic matter, nitrogen removal via combination of nitrification-denitrification [10]. Microbes may also perform a recently discovered process-nitrite/nitrate-dependent anaerobic methane oxidation (N-damo) [29]. Microorganisms also play an important role in phosphorus removal in CWs [30]. They change the pH of the environment and increase the sorption surface of the “matrix”, which is composed of organo-mineral films on plants and soil surfaces. As a result, the differences in treatment efficiencies between CWs can be explained by variation in microbial community compositions [31]. Thus, to improve the design and performance of a CW, it is necessary to understand and manage the structure and diversity of microbial communities. High-throughput sequencing of environmental DNA (metabarcoding) enables us to accurately analyze microbial community composition and diversity, as well as to predict its functional profile. However, this method is still rarely used to determine microbial communities of CWs.

The composition of pollutants in the influent is very diverse. For effective purification, CWs include several subsystems (functional zones) where contrasting biochemical processes occur. CWs usually include zones with aerobic and anaerobic conditions, zones of sludge dewatering, and buffer zones for wastewater with variable hydraulic loading [32,33]. Since these areas are characterized by different particle-size composition, physicochemical conditions, and vegetation, one can expect the presence of separate microbial communities in different zones of a CW. On the other hand, it is still unknown whether short retention time is sufficient for the formation of a specific microbial community in a CW.

In the present study, we analyzed spatial changes in microbial communities along different functional zones of a free-water surface wetland treating river and storm water. The CW is located on the territory of Moscow, Russia. The average retention time in this CW is about 6 h, which is much less than in European and American CWs (one day versus three to five days). We hypothesized that zones with distinct conditions (e.g., aeration, physical and chemical properties of sediment matrix) would be characterized by different microbial composition and diversity. We also aimed to test the link between microbial composition and pollutant removal efficiency for the considered zones of the constructed wetland.

## 2. Materials and Methods

### 2.1. Constructed Wetland Design and Operation

The samples of sediments were collected in August 2016 from an operating free-water surface wetland, located in Moscow, Russia (55°42′13″ N; 37°36′7″ E). The climate is moderately continental, with warm summers and moderately cold winters. The annual mean total precipitation is 708 mm. The average annual temperature is 6.8 °C. The CW started its operation in 2001. The facility was designed to treat wastewater formed as a result of mixing a part of the runoff of the regulated river Krovyanka with the diffuse runoff from the roadbed named the Third Ring Road. There is a cemetery, a large highway, hypermarkets, shopping and entertainment center, car wash, as well as residential areas with multi-storey buildings and office centers located near the studied constructed wetland. There is a cogeneration plant nearby, where warm wastewaters can also flow into the wetland. The wastewaters entering the CW are characterized by heterogeneity in physical and chemical characteristics (Table 1). 

The studied wastewaters are generated from diffuse and local urban surface waters, including industrial effluents. The first and the main difference of the studied wastewater from other wastewaters in Moscow consists in its higher temperature all year round. This is due to the influent from a thermal power plant located two kilometers upstream of the Krovyanka River. The second important feature of the influents is their heterogeneous composition throughout the year. Variations in dissolved O_2_, pH, electrical conductivity, suspended substances and chemical oxygen demand (COD) can be ranged up to 500% of the mean values. The high COD values that are periodically observed in the studied wastewater indicate volley discharges of hardly oxidizable organic matter, most likely oil products which enter the effluent from several car washes and are discharged into the Krovyanka River upstream. On the other hand, the contents of total nitrogen and nitrogen compounds correspond to the average values wastewater in Moscow. Compared to the studied constructed wetland, the influents entering a CW in Spain are characterized by a higher temperature, lower COD and NH_4_^+^ values; but pH, oxygen, and TSS values are similar to those in our study [25]. In a Chinese CW, the influents have higher temperature, but lower NO_3_^−^ content; NH_4_^+^ and NO_2_^−^ are the same as in our study [34].

**Figure 1:**
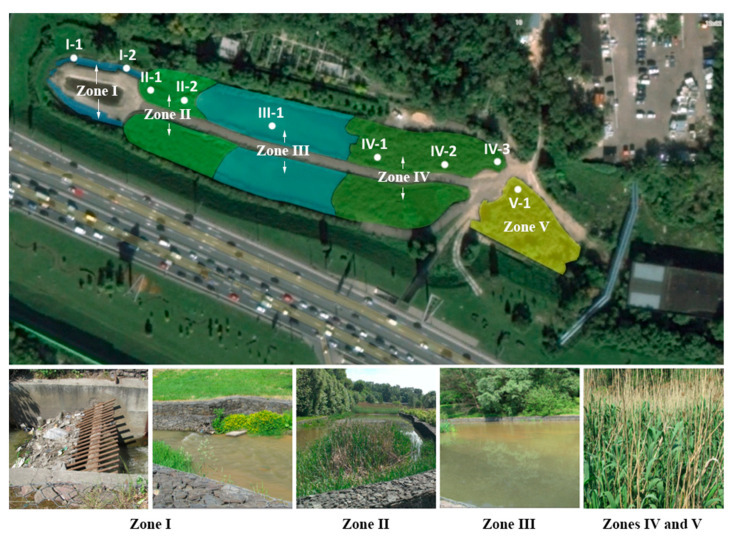
Scheme of the constructed wetland studied. Zone I represents sand traps and grids; zone II—phytofilters; zone III—sedimentation tank; zone IV—phyto-treament; zone V—additional phyto-treatment. One or several sub-zones were selected in each functional zone.

The total area of the CW is 5925 m^2^ and the volume is 7445 m^3^ (Figure 1). The total length of the CW is 290 m. The average consumption of water entering the CW is 34.000 m^3^ per day, with the average time spent of six to seven hours. The walls of the facility consist of gabions (cages filled with rocks). The wetland is divided into two equal channels (left and right) and consists of an inlet structure, large debris filtration grids, two 1.5 m deep sand traps, two 2 m deep ponds, two shallow areas with 0.3 m deep vegetation, a 1 m deep outlet channel, and a temporary storage area for sediments. The vegetation of the CW is represented by *Typha* sp. and *Phragmites* sp. The treated wastewater flows into the Chura River. According to the specific processes of the site, we have identified five functional zones within the CW: (1) sand traps and grids; (2) phytofilters; (3) sedimentation tank; (4) phyto-treatment; and (5) additional phyto-treatment areas (Figure 1; Table 2).

### 2.2. Organic Contaminants Analysis

In the CW sediments, the contents of three groups of pollutants were measured: (1) hydrocarbons, including petroleum products (HC): medium boiling fraction, high boiling fraction and total petroleum products; (2) phthalic acid esters (phthalates); (3) polycyclic aromatic hydrocarbons (PAHs). For this purpose, the mixed samples of sediments were placed in 500 mL glass containers with a tightly screwed lid. Then the samples were dried at 80 °C. Stones, roots, and anthropogenic inclusions were taken out, and the rest was homogenized and passed through a sieve with a pore size of 1 mm. Prior to measurements, the samples of bottom sediments were stored in a freezer at −20 °C.

Organic compounds were extracted from the bottom sediments samples by subcritical solvent extraction using the ASE 200 automated accelerated extraction (Dionex, Sunnyvale, CA, USA). Specifically, 2 g of each sample was ground with 2 g of prepared silica sand; the resulting mixture was poured into the cell for accelerated automatic extraction with a volume of 11 mL with a cellulose filter placed on the bottom. Extraction conditions were the following: 100 °C, 1500 psi, preheating time was 5 min, exposure time—5 min, washing volume—120%; nitrogen purge time—60 s; the number of cycles—2. Two sequential treatments of the samples were carried out using a mixture of chloroform:methanol (3:1 by volume) as an extractant, then chloroform. The extracts obtained after ASE 200 were freed from water, and were then placed in a freezer (−20 °C) for 1.5 to 2 h. Then, the extracts were combined and dried on a rotary evaporator at a temperature not exceeding 40 °C; the residue was redissolved in 10 mL of chloroform. The aliquots were taken from the obtained extract to purify the samples.

Purification and fractionation of the samples were carried out by the adsorption method. The samples were added to a prepared column filled with 4 g of alumina (activity grade II), after which the hydrocarbon fraction (HC) and the fraction of weakly polar ethers were successively eluted with 10 mL of hexane and 10 mL of a mixture of hexane: chloroform (1:1 by volume). The eluates were collected in different tubes. Both fractions were evaporated on a rotary evaporator to a volume of 0.25 mL, quantitatively transferred to a chromatographic vial and supplemented with hexane to 1 mL.

To determine the PAHs content in the samples, the extracts were purified on a prepared solid-phase extraction cartridge with Diapak-S sorbent (BioChemMak, Moscow, Russia). The desired fraction was eluted with benzene. The eluate was distilled off and redissolved in 1.5 mL of acetonitrile for a quantitative analysis. Identification and quantification of organic compounds in the samples were performed by capillary gas-liquid chromatography on an Agilent 6890 N gas chromatograph (Agilent Technologies, Santa Clara, CA, USA) with a quadrupole mass-selective detector MSD5973 N (Agilent Technologies, USA) and a 30 m × 0.25 DB1-ms column mm × 0.25 μm (Agilent Technologies, USA).

The compounds were identified by retention times on chromatograms and mass spectra (NIST/EPA/NIH 08 Mass Spectral Library, NIST Mass Spectral Search Program ver. 2.0 f). The quantification of compounds was carried out by the dominant fragment ions (base peaks): m/z 57—hydrocarbons, m/z 149—phthalic esters. As calibration standards, substances of guaranteed purity and non-polar fractions of oil products—diesel fuel L 0.5–62 and motor oil SAE 15 W-40—were used. A quantitative mixture of C10-C34 Connecticut n-Hydrocarbon Mix n-alkanes (Supelco, Switzerland) was used as an external standard for detector calibration.

The PAHs were measured by high pressure reverse phase chromatography on an Agilent 1100 liquid chromatograph (Agilent Technologies, USA) with a fluorimetric detector and a Diasfer 110-C18 column, 5 μm, 4.0 × 250 mm (BioChemMak, Moscow, Russia). As calibration standards, a set of standard PAH samples in acetonitrile SOP 0118-03 ER-PAH were used.

### 2.3. Particle-Size Distribution Analysis

Laser analyser Microtrac Bluewave (Microtrac, York, PA, USA) was used to determine the particle size distribution (PSD) of the samples. The speed of circulation was 50% of maximum. Calculation of the results was made using the following parameters: particles were described as absorbing (absorption coefficient—1) and of irregular shape, refractive index of distilled water—1.33. Equipment software takes into account that the refractive index of absorbing particles does not significantly affect the results. The selected parameters were in agreement with the previous studies [35]. Two types of pre-treatment were used: with and without sonication. In the first case, a sample aliquot (a few millilitres) was placed directly on the sample dispersion controller unit of an analyser and was then analysed. In the second case, the samples were previously prepared by a horn type ultrasonic disruptor (Stepped Solid Horn 1/2″, Digital Sonifier S-250 D, Branson Ultrasonics, Danbury, CT, USA). Ultrasonic power was calibrated calorimetrically. Ultrasonic dispersion energy was 450 J mL^−1^.

### 2.4. Total DNA Extraction and Amplicon Sequencing 

Total DNA was extracted and purified from 0.2 g of each sediment sample in three replicates using the MagNA Pure Compact Nucleic Acid Isolation Kit I (Roche, Basle, Switzerland) according to the manufacturer’s protocols. The lysis of the cells and tissues was performed using MagNA Pure Bacteria Lysis Buffer (Roche, Basle, Switzerland). Mixed DNA samples were then prepared from three replicates per sample. NanoDrop ND-1000 (Thermo Fisher Scientific, Waltham, MA, USA) was used to assess DNA concentration and detect possible contaminations (A260/A280 ratio). The extracted DNA samples were stored in −20 °C until further analyzes.

The V3–V4 region of 16S rRNA was amplified using universal primers 341 F (5′-CCTACGGGNGGCWGCAG-3′) and 805 R (5′-GACTACHVGGGTATCTAATCC-3′) [36,37]. This primer pair has been chosen because they are the most effective to cover different bacterial groups [38] and are also commonly used in recent studies on the CW microbiome [39,40,41,42,43]. We added this explanation to the MS (L. XXX). Further preparation of the 16S rRNA gene libraries was carried out as described in the MiSeq Reagent Kit Preparation Guide (Illumina, San Diego, CA, USA). The sequencing of 16S rRNA gene amplicons was performed on an Illumina MiSeq platform using MiSeq^®^ Reagent Kit v2 (500 cycles) with paired-end 2 × 251 cycle sequencing mode. The bcl2 fastq v2.20 Conversion Software (Illumina) was used to demultiplex data and convert BCL files to standard FASTQ file formats. 

### 2.5. Statistics and Bioinformatics

The pair-end reads were filtered and merged using MeFiT [44]. The sequences were grouped into operational taxonomic units (OTUs) according to 97% similarity in the nucleotide composition using the RDP database with the RDP classifier [45]. The RDP classifier’s confidence threshold was set to 90%. The data was normalized by the total read count. The singletons (the OTUs containing a single sequence) were removed as well as the chloroplast and mitochondrial 16S rRNA sequences.

Microbial diversity was estimated using the Shannon index and the total number of the detected genera. Bray-Curtis dissimilarity was used to explore the variation in prokaryotic community structures among all the samples. Non-metric multidimensional scaling (NMDS) was performed on distance matrices and the coordinates were used to draw 2D graphical outputs. Alpha- and beta-diversity indexes were aligned based on the number of reads per sample. The heatmaps and dendrograms were generated using the ggplot2 R package. The functional gene profiles were predicted based on 16S rRNA data using the R package Tax4 Fun [46].

## 3. Results

### 3.1. Chemical Composition of Organic Pollutants in the Sediments

**Figure 2:**
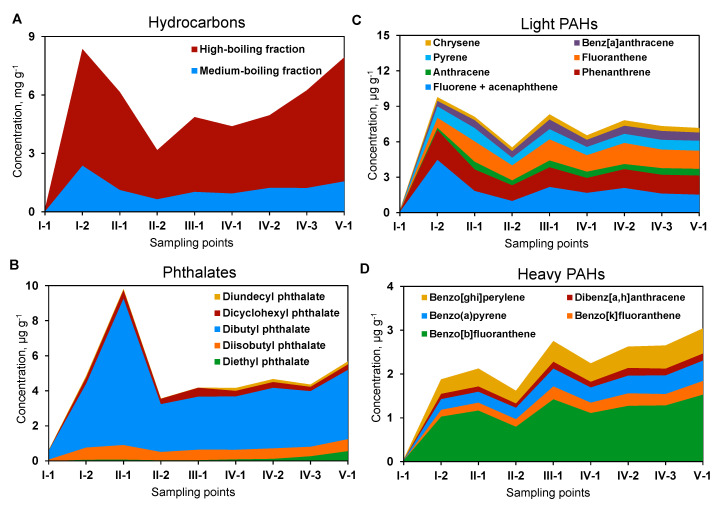
Chemical composition of organic pollutants in sediments of different zones of the constructed wetland. (**A**) hydrocarbons; (**B**) phthalates; (**C**) light polycyclic aromatic hydrocarbons (PAHs); (**D**) heavy PAHs.

The identified concentrations of organic pollutants in the sediments resulted from a combination of two processes: sedimentation (increase of concentration) and degradation (decrease). The high boiling fraction prevailed among hydrocarbons in the CW sediment (71.5–81.7%), however, the distribution of both fractions in different CW zones had a similar trend (Figure 2A, Table S1). Zone I (sand traps) had the lowest concentrations of hydrocarbons (medium boiling fraction of 32 μg g^−1^, high boiling fraction of 115 μg g^−1^). A sharp increase in the concentration of hydrocarbons, especially of the high boiling fraction, was revealed at the end of zone I. There was a two to four times decrease in the concentrations of both fractions in zone II-2 (phytofilters). In the sedimentation tank (zone III), the hydrocarbon content increased again and continued to increase in subsequent areas, reaching the highest values in the outlet area of the CW (V-2) (Figure 2A, Table S1).

The following phthalates were detected in CW sediments: diethyl phthalate, dibutyl phthalate, diisobutyl phthalate, dicyclohexyl phthalate, and diundecyl phthalate. Dibutyl phthalate was characterized by the highest concentrations (0.518 mg g^−1^ in zone I-1 and 3.960 mg g^−1^ in zone V-2), while the contents of the other phthalates did not exceed 0.1 mg g^−1^ (Figure 2B). The concentrations of all phthalates increased sharply when passed through the sand trap (zones I-1 and I-2), but then decreased when passed through phytofilters (zone II). A subsequent slight increase in concentrations of phthalates was found in the sedimentation tank (zone III). In the remaining areas of the CW, the contents of phthalates changed insignificantly, except for diethyl phthalate which increased to 0.561 mg g^−1^ in the outlet area of the CW (Figure 2B, Table S1).

Altogether, eight light polycyclic aromatic hydrocarbons (PAHs) (Figure 2C) and five heavy PAHs (Figure 2D) were identified in the CW sediments. The concentrations of all the analyzed PAHs were the lowest in zone I-1 of the CW and then increased sharply in zone I-2. The concentrations of PAHs decreased after passing through the phytofilters. The concentrations of light and heavy PAHs increased again in zone III (sedimentation tank), while in the phyto-treatment areas their contents did not almost change (Figure 2C,D).

### 3.2. Particle-Size Distribution (PSD) of the Sediments

**Figure 3:**
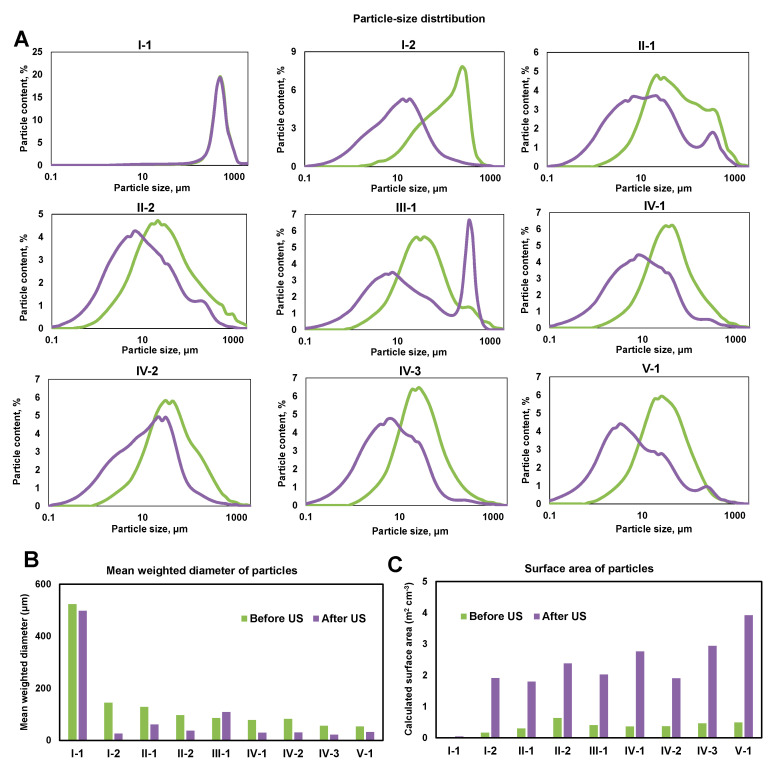
Physical composition of particles in sediments of different zones of the constructed wetland. (**A**) particle size distribution before and after ultrasonic; (**B**) mean weighted diameter of particles; (**C**) surface area of particles.

The particle size distribution of the sediments in different CW zones varied widely from hundreds of micrometers to several millimeters (Figure 3A). Most of the sediment samples were characterized by a monomodal distribution (Figure 3A). At the beginning of the sand trap (zone I-1), the sediments were represented only by sand particles. The particle size distribution before and after sonication was the same; the average particle diameter was about 500 μm, and the surface area was less than 0.05 m^2^ cm^−3^ (Figure 3A,B). At the end of the sand trap (zone I-2), the size of the sediment particles changed (Figure 3A). The particles larger than 1000 μm were absent in the sediment of this zone, however, there was sludge with a particle size of mainly 200 to 350 μm. A fraction of 10 to 20 μm prevailed after sonication (Figure 3A). The particle surface area was 0.2 m^2^ cm^−3^ and 1.9 m^2^ cm^−3^ before and after sonication, respectively (Figure 3B,C). 

At the beginning of the phytofilter zone (II-1), particles with a size of 20 to 50 μm before sonication and 5 to 30 μm after sonication dominated; a maximum was also observed for particles with diameters of 300 to 350 μm. At the end of zone II, there was a slight increase in the share of more dispersed particles. In the sedimentation tank (zone III), the particle size distribution before sonication was similar to the previous zone, and two maximums of 5 and 350 μm were observed after sonication. In the phyto-treatment zone (IV), particle fractions of 30 to 50 μm and 10 to 20 μm prevailed before and after sonication, respectively. In zone V-1, particles of 15 to 50 μm dominated before sonication, while particles of 5 μm were the most abundant after sonication (Figure 3A). In the outlet zone of the CW after dispersion, 13% of the particles were represented by a fraction of <1 μm, and the specific surface area of the particles was 3.9 m^2^ cm^−3^ (Figure 3B,C).

### 3.3. Microbial Community Composition and Diversity

The difference between the lowest and highest values of DNA concentrations was 20 times. The lowest DNA concentrations, 2.4 and 4.8 ng µL^−1^, respectively, were revealed in the inlet zone of the CW (I-1) and in the sedimentation tank (III). Zones II-2 and IV were characterized by the highest DNA concentrations (38.3 and 47.5 ng µL^−1^, respectively) (Table S2).

**Figure 4:**
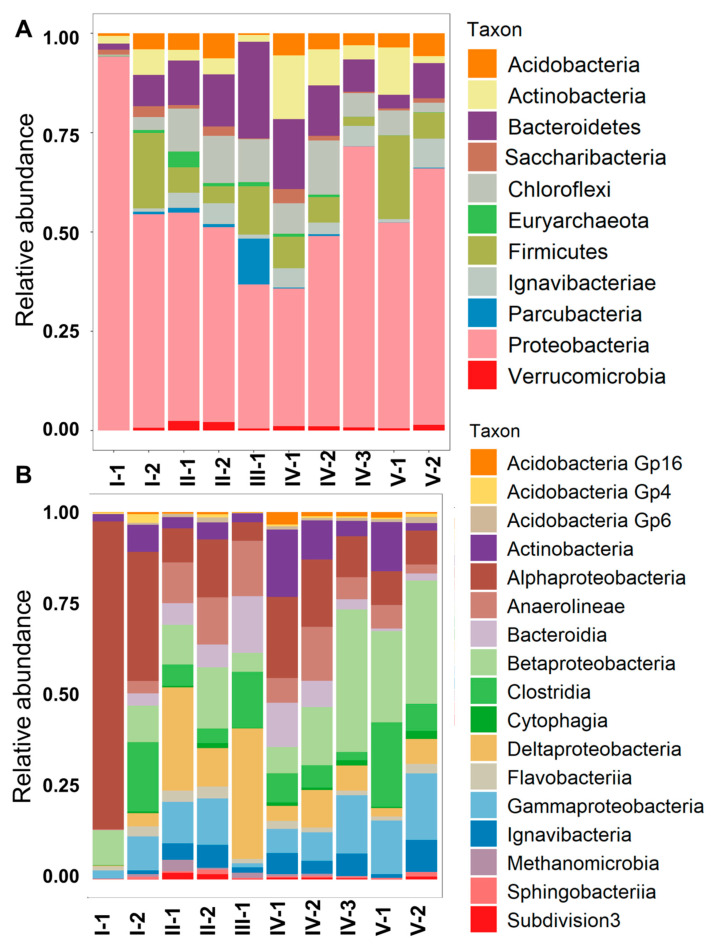
Relative abundances of microbial taxa of considered functional zones of the constructed wetland at the phylum (**A**) and the class (**B**) levels. The data are presented for phyla and classes with abundance of more than 0.5%.

In general, the number of obtained nucleotide sequences ranged from 79,565 to 199,427 reads per sample. Representatives of 1013 genera belonging to 79 classes and 46 phyla were identified in the studied CW. The number of sequences ranged from 22,277 to 85,637 (the average for all the samples was 45,943) (Table S2). The sediments of different zones within the CW were characterized by a clear spatial heterogeneity of microbial communities (Figure 4A). The dominant phyla in microbial communities of the CW were Proteobacteria, Firmicutes, Bacteroidetes, Chloroflexi, and Actinobacteria. Representatives of Acidobacteria, Ignavibacteriae, and Verrucomicrobia were also identified in all the CW zones, but their relative abundances did not exceed 1–2% in most of the zones. Proteobacteria absolutely dominated in the inlet zone of the CW (I-1) with a relative abundance of 90.1%, as well as at the ends of phyto-treatment zones IV-2 and V-2 with shares of 62% and 55%, respectively (Figure 4A). The lowest share of Proteobacteria (16.6%) was detected in anaerobic zone III. In the remaining zones, the relative abundance of Proteobacteria varied in a narrow range of 33% to 37%. Firmicutes were almost absent in the inlet zone of the CW (I-1), however, in subsequent areas they were characterized by a high relative abundance, with a maximum in zones I-2 and V-1 (26.2% and 36.7%, respectively). Bacteroidetes and Chloroflexi had very similar distribution trends in different areas. The lowest shares of these groups were found in the inlet and outlet zones of the CW, while the highest relative abundances (17–26%) were present in the intermediate zones of the CW (from II-2 to IV-2). Zone II was characterized by a higher proportion of Verrucomicrobia and methanogenic Euryarchaeota. Parcubacteria had higher relative abundance in zone III, while Actinobacteria and Acidobacteria were more abundant in zone IV (Figure 4A).

A total of 17 classes with a relative abundance of >0.5% were identified (Figure 4B). The dominant classes in the studied CW were Alpha-, Beta-, Gamma-, and Deltaproteobacteria, as well as Anaerolineae, Bacteroidia, and Clostridia. Across the CW, the relative abundance of Alphaproteobacteria decreased from 83.9% in zone I-1 to 6.9% in zone V-2, while the share of Betaproteobacteria increased from 9.1% in I-1 to 24.0% in V-2. In zone II (phytofilters), the relative abundances of Deltaproteobacteria (17.9%), Anaerolineae (6.9%), and Gammaproteobacteria (6.6%) increased as well. Microbial community of zone III (sedimentation tank) was characterized by a sharp decrease of all classes of Proteobacteria, with the exception of Deltaproteobacteria (8.4%), as well as by the predominance of Clostridia (16%), Bacteroidia (8.9%), and Anaerolineae (5.5). Betaproteobacteria and Clostridia prevailed in zone V (Figure 4B).

**Figure 5:**
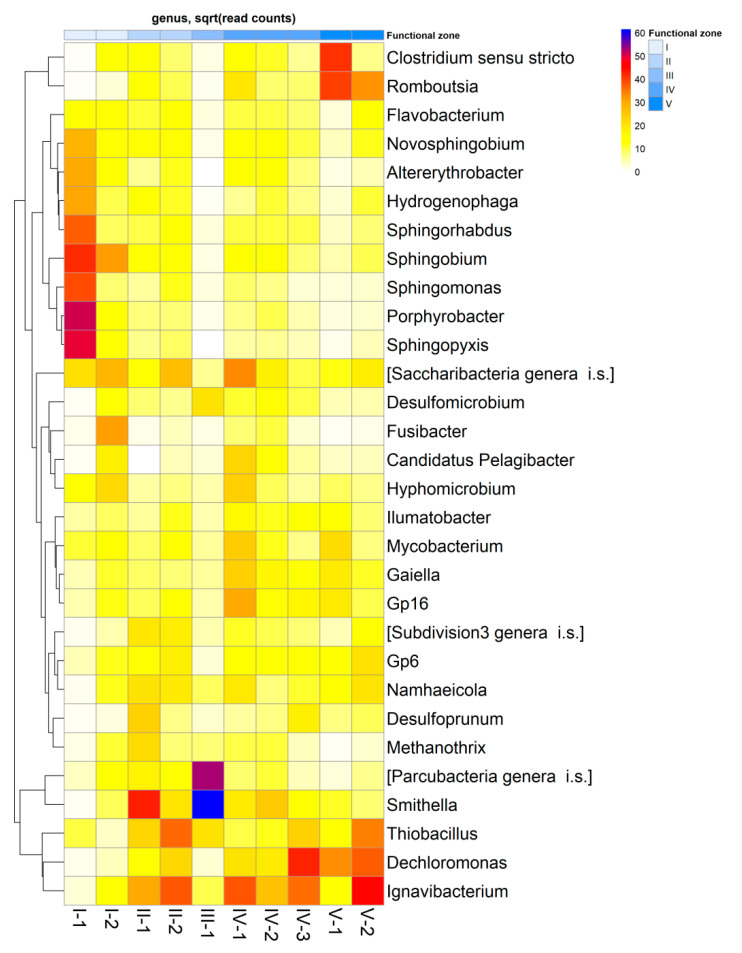
Relative abundances of top 30 microbial taxa of considered functional zones of the constructed wetland at the genus level. The data is given as square roots of the read counts. Abundances of prokaryotic genera increase in a row: white–yellow–red–violet–dark blue.

Altogether, 41 prokaryotic genera with a share of >0.5% were identified. Microbial composition in the inlet zone of the CW (zone I-1) was sharply different from other CW zones (Figure 5). Porphyrobacter (3.5%), representatives of Sphingomonadaceae family (Sphingobium, Sphingopyxis, Sphingomonas, Sphingorhabdus, and Novosphingobium were 21.4% in total), Hydrogenophaga (4%), and Altererythrobacter (1.2%) were dominant genera in this zone. The relative abundances of all these microbial genera were the highest in the inlet zone of the CW and significantly decreased in subsequent areas. In zone I-2, Sphingobium (2.6%), Fusibacter (5.1%), Proteiniclasticum (3.2%), and Acinetobacter (2.2%) were the most dominant genera within a microbial community. In the phytofilter zone, the shares of Smithella (from 0.1% in zone I-2 to 2.2% in zone II-1), Ignavibacterium (from 0.2% to 1%) and Methanothrix (from 0.1% to 0.9%) increased sharply. In terms of genera, the microbial community of the sedimentation tank (zone III) with anaerobic conditions was strongly different from other zones of the CW. In the sedimentation tank, Smithella (4.2%), Proteiniclasticum (3.5%), Parcubacteria (6.7%), Macellibacteroides (2.3%), and Desulfomicrobium (0.9%) were the dominant genera. In the phyto-treatment zone, Ignavibacterium, Saccharibacteria, Acidobacteria Gp16, and Smithella had the highest relative abundances within a microbial community, while at the end of this zone the shares of Dechloromonas (6.1%) and Thiobacillus (1.2%) strongly increased. At the beginning of the outlet zone (V-1), Clostridium (13.4%), Romboutsia (9.7%), and Dechloromonas (3%) were the dominant genera. In the outlet of the CW (V-2), Dechloromonas (4.6%), Ignavibacterium (2%), Thiobacillus (2.3%), and Romboutsia (7.7%) were the major microbial groups in the microbial community (Figure 5).

Relative abundances of many microbial taxa were strongly correlated to the physical characteristics of particles in the sediments (diameter and surface area of particles, the proportion of different size fractions) (Table 3). Most of bacterial phyla (Acidobacteria, Bacteroidetes, Chloroflexi, Firmicutes, and Verrucomicrobia) were positively correlated to the surface area of particles and to the particles of average (1–100 µm) and low (<1 µm) diameters (Table 3). Almost all classes and genera of Acidobacteria were confined to small-diameter particles. Alphaproteobacteria was the only bacterial class that was very strongly correlated to the large particles of 100 to 1000 µm (r = 0.93) and >1000 µm (r = 0.87) diameters. At the genus level, most of these Alphaproteobacteria belong to the Sphingomonadales order: *Altererythrobacter*, *Novosphingobium*, *Sphingobium*, *Sphingomonas*, *Sphingopyxis*, and *Sphingohabdus* (Table 3). 

### 3.4. Diversity and Community Clustering

**Figure 6:**
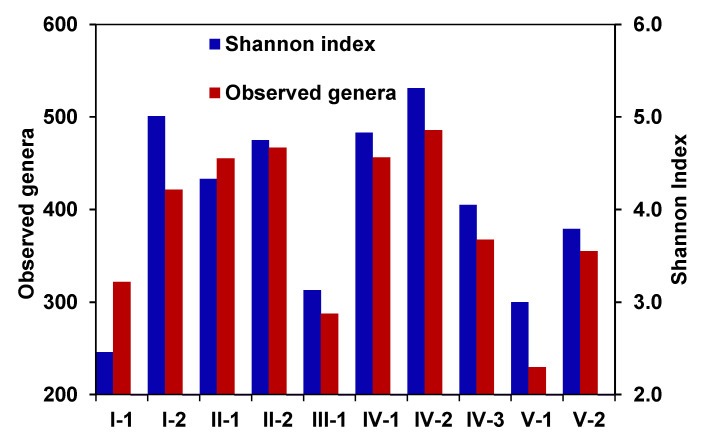
Alpha diversity (numbers of observed genera and Shannon indexes) of microbial communities of different functional zones of the constructed wetland.

According to the microbial diversity indexes, the zones within the CW were clustered into two groups. The microbial communities at the end of zone I, as well as in zones II and IV were characterized by a higher Shannon index (4.2–4.9) and a higher number of the detected genera (433–531) (Figure 6). Microbial diversity in the inlet and outlet zones of the CW, as well as in zone III with anaerobic conditions was significantly lower: the Shannon index ranged from 2.9 to 3.5, while the number of taxa ranged from 246 to 379 (Figure 6).

**Figure 7:**
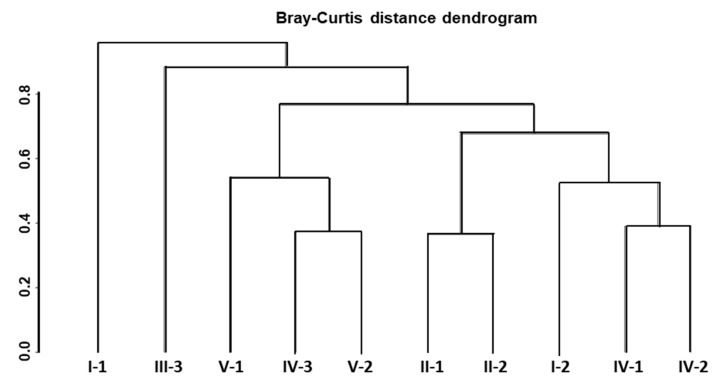
Microbiome clustering dendrogram of different functional zones of the constructed wetland based on Bray−Curtis (BC) distance matrix.

A dissimilarity analysis based on Bray−Curtis metrics allowed us to assess the extent of differences between microbial communities of different CW zones and separate them on several clusters (Figure 7). Microbial communities of I−1 (the sand trap in the inlet zone of the CW) and III−1 (sedimentation tank) zones were characterized by the highest differences compared to other zones (Figure 7). The microbiomes of II−1 and II−2, IV−1 and IV−2, as well as the final three zones corresponding to the final wastewater purification stages of the CW (IV−3, V−1 and V−2) were divided into separate groups.

### 3.5. Prediction of Microbial Functional Profiles

**Figure 8:**
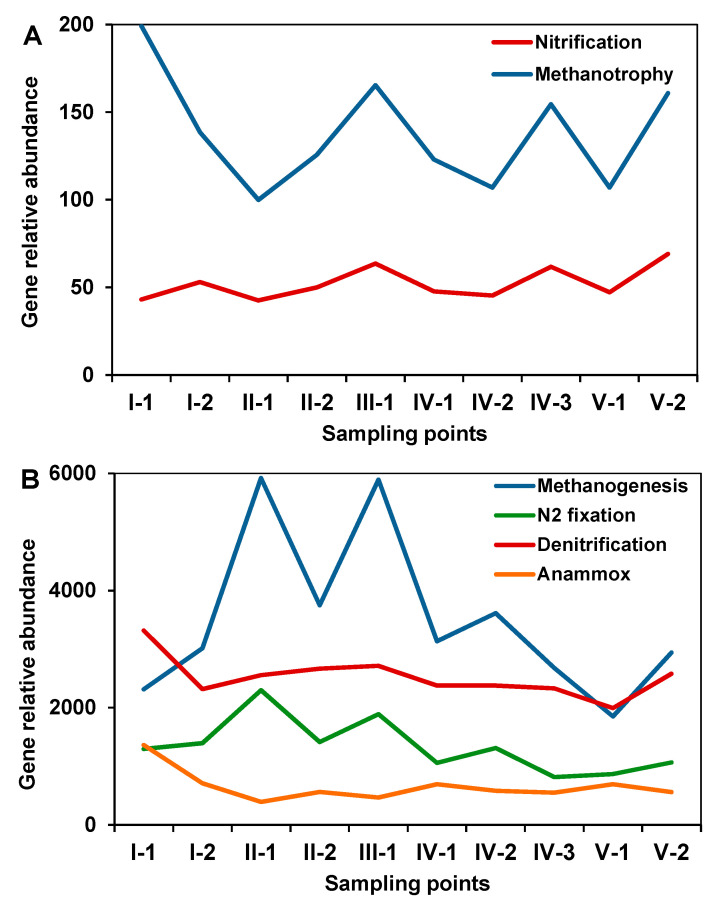
Predicted microbial functional profiles of different zones of the constructed wetland. Processes were divided on two groups: (**A**) aerobic, low abundant processes; (**B**) anaerobic, abundant processes.

Using the Tax4 Fun software package, the functional gene profiles of microbial communities of different CW zones were predicted. The functional genes were grouped according to their certain microbiological processes-aerobic (nitrification, methanotrophy) and anaerobic (nitrogen fixation, denitrification, anammox, methanogenesis) (Figure 8A,B). The relative predicted abundances of genes responsible for anaerobic processes were 20 to 30 times higher compared with the genes associated with aerobic processes. The share of the predicted functional genes associated with nitrification was very small and practically did not change in different zones of the CW. Zone I (sand trap) was characterized by the largest share of the predicted functional genes associated with methanotrophy, anammox, and denitrification. Most of the functional genes responsible for nitrogen fixation were detected in zone II (phytofilters). The phytofilters and anaerobic sedimentation tank were also characterized by the highest predicted relative abundances of genes responsible for methanogenesis (Figure 8B).

### 3.6. Disinfection in the CW

**Figure 9:**
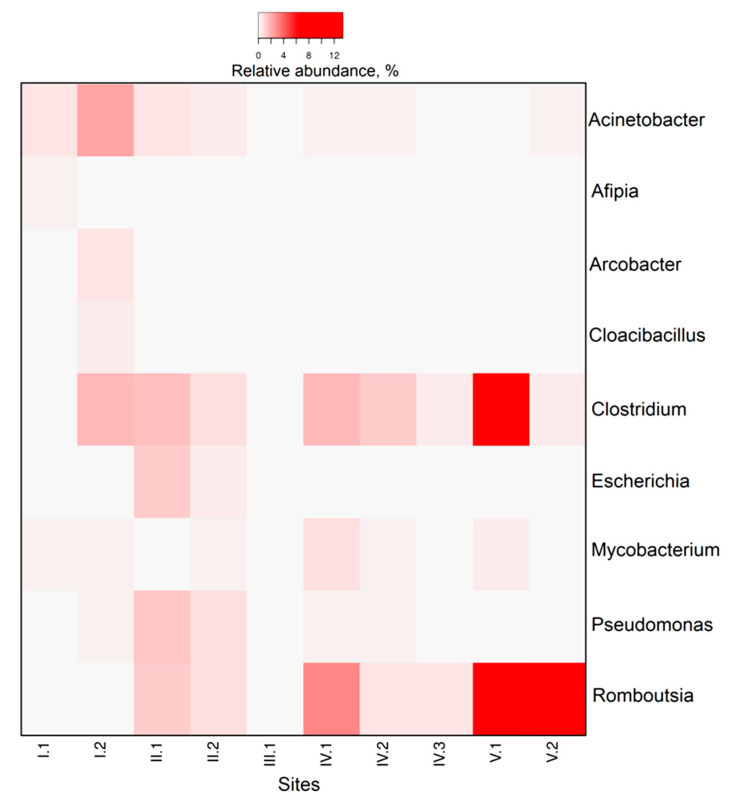
The distribution of potentially pathogenic microbial genera in different zones of the CW. The pathogenicity of *Romboutsia* has not yet been confirmed, but they were included to the figure as they are typical human gut inhabitants.

Among the potential human pathogens, seven genera were characterized by a share of more than 0.5% in at least one of the CW zones: Acinetobacter, Afipia, Arcobacter, Cloacibacillus, Escherichia, Mycobacterium, and Pseudomonas (Figure 9). Typical gut genera Clostridium (some strains may also be pathogens) and Romboutsia (pathogenicity has not yet been proven) have also been identified. The relative abundances of seven major human pathogens significantly decreased from the inlet (1.2–3.7% in total) to the outlet zone (0.5%) (Figure 9). On the contrary, the shares of Clostridium and Romboutsia increased in the outlet zone of the CW up to 13.4% and up to 9.7%, respectively.

## 4. Discussion

### 4.1. Functional Zones of the Constructed Wetland Are Characterized by Contrasting Physicochemical Properties of the Sediments

The conducted study showed the spatial heterogeneity of physicochemical and microbiological properties of the sediments in the CW. The identified concentrations of organic pollutants in the sediments resulted from a combination of two processes—sedimentation/sorption and biochemical degradation of pollutants. While sedimentation leads to an increase in the concentration of pollutants, decomposition decreases it. Among all the pollutants studied, only phthalates decomposed in the CW at such a short retention time of wastewater treatment. The rest of the pollutants were partially decomposed in different zones, but the main mechanisms for their removal from wastewater were sorption and sedimentation.

Since the sorption of organic pollutants was the most important process of their accumulation in the constructed wetland, the specific surface area of mineral particles in the sediments plays an essential role in the removal of pollutants from wastewater. The specific surface area of particles in the sediments increased from the inlet to outlet zone within the CW. Sorption and sedimentation of suspended substances containing hydrocarbons, phthalates, and PAHs were not active in the sand trap area. This resulted in low concentrations of all the analyzed pollutants at the inlet of the CW. In the sand trap zone, the sediments were represented by coarse sand particles with a size of about 500 μm. These particles have a small surface area, hence low sorption capacity.

Environmental conditions and processes in the phytofilter zone were mostly determined by the presence of vegetation and associated microorganisms. The phytofilter zones were characterized by the highest rate of organic pollutant sorption and decomposition. The particles in the phytofilter sediments were characterized by the highest surface area which contributed to the high sorption of pollutants. The formation of organo−mineral aggregates in this zone could be explained by plant and microbial activity. Two phyto−treatment zones of the CW had a crucial role in wastewater purification. It is well known that plants facilitate the elimination of many pollutants in CWs, such as organophosphorus pesticides [18] or pharmaceutical compounds [20].

In the phyto−treatment zones, specific organic particle aggregates similar to activated sludge flocs were identified in the sediments. Activated sludge flocs are heterogeneous structures formed by microorganisms, inorganic particles, proteins, and polysaccharides [47,48]. These floc aggregates were stable and existed in the root zone of the phyto−treatment zones. In our study, the size of floc aggregates was 15 to 50 μm, but after sonication and destruction of polysaccharide bonding we identified that floc aggregates consisted mostly of mineral particles of 5 μm. This correlates with a recent study, which showed that 90% of activated sludge particles were represented by a fraction of 1 to 10 μm [49]. 

During floc sedimentation, dispersed material such as bacterial cells and small flocs becomes attached to the floc surface [50]. Microorganisms in the outer parts of the flocs are loosely bound to the floc matrix and can be easily eroded from the surface when exposed to shear [51].

### 4.2. Functional Zones of the Constructed Wetland Are Characterized by Distinct Taxonomic and Functional Microbial Profiles 

Treatment efficiency of CWs can be explained by variation in microbial community composition [31]. Unlike soils or most other environments, the ecological conditions in wetlands are highly spatially and temporally variable. This is due to the short retention time of wastewater which has temporal variation in its composition. Some of the microorganisms can be washed away by wastewater; some can be introduced with wastewaters. CW ecosystems are also characterized by a constant supply of organic substrates from wastewater for microorganisms. As a result, the overwhelming majority of microorganisms in CWs are metabolically active. This is strongly different from soil ecosystems where most microorganisms are metabolically inactive [52,53]. 

One of the most important and unexplored features of CWs is the formation time of a specific microbial community, as well as dependence of the stability of the community structure on hydraulic retention time. Despite short retention time (6 h) of wastewater in the given CW, we revealed spatial changes in microbial communities of the sediments in different functional zones. The degree of spatial variability of the CW microbiome was controlled mainly by the movement of wastewater from the inlet to the outlet zones of the CW, as well as by the environmental conditions and vegetation cover that were performed for different functional zones of the CW. Moreover, we found that many microbial taxa were strongly correlated to the physical characteristics of particles in the sediments. Almost all classes and genera of Acidobacteria were confined to small−diameter particles (<1 µm). On the other hand, most of Alphaproteobacterial genera belonging to Sphingomonadales order were strongly correlated to large particles (>100 µm). It is suggested that there is an ecological relationship between Acidobacteria and Proteobacteria because they are often observed to be intimately associated (more often, negatively correlated) with each other in the environment, and may influence each other’s position in the community [54]. Alphaproteobacteria are considered as copiotrophs [55,56], while Acidobacteria are often considered as an oligotrophic group [54,55]. In addition, the presence of only one or two copies of 16S rRNA genes in the genomes of Acidobacteria was revealed, suggesting lower growth rates, which often correlates with oligotrophy [54,57]. The association of Acidobacteria with particles of <1 µm also means that this microbial group is characterized by cells of small size.

A strong (20−fold) variation in DNA concentration was observed in different zones of the CW. DNA concentration can be an indicator of microbial biomass; in soils, the ratio between DNA yields and microbial biomass C can be about five [58]. In this case, microbial biomass C in the sediment of the CW ranged from 6.1 to 121 µg g^−1^ moist sediment, which is equal to C−poor soils. The lowest microbial biomass C was revealed in the inlet zone of the CW and in the sedimentation tank, while zones of phytophilters and phyto−treatment were characterized by the highest microbial biomass.

The microbiome of the inlet zone was the most different from those in other zones of the CW and was characterized by the dominance of microbial genera belonging to the Sphingomonadaceae family: Sphingomonas, Sphingobium, Sphingopyxis, Novosphingobium, and Porphyrobacter. The representatives of Sphingomonadaceae are common inhabitants of soil and rhizosphere of plants [56]. Sphingomonadaceae can utilize hardly decomposable aromatic compounds, which makes these bacteria of interest to environmental remediation [59]. Sphingomonas are found in the habitats contaminated with pesticides, polychlorinated biphenyls, creosote, and pentachlorophenol [59]. Representatives of the genus Sphingobium are capable of decomposing aromatic (including chlorine−containing) compounds, phenols (e.g., nonylphenol and pentachlorophenol), herbicides and polycyclic aromatic hydrocarbons [60]. Representatives of the genus Sphingopyxis are able to utilize chlorophenol, styrene, and phenylacetic acid [61,62], while Novosphingobium utilizes phenol, nitrophenol, aniline, phenanthrene, respectively [63]. Porphyrobacter is able to decompose biphenyl and dibenzofuran [64]. Sphingomonadaceae are chemoheterotrophs and strict aerobes. Therefore, a decrease in its relative abundances from the inlet to the outlet zone of CW may indicate a gradual reduction in pollution and aeration of wastewater when passing through the CW zones. Furthermore, the sand trap was characterized by the highest relative abundance of methanotrophic genes, as well as by a low share of genes responsible for methanogenesis. Altogether, it indicates the predominance of aerobic conditions and processes in the inlet zone of the CW.

The phytofilter sediments had a greater microbial biodiversity than the grid area. It was shown that the Shannon index could serve as a bioindicator of pollutant removal efficiency in the CW [65]. The microbiome of the phytofiler area was represented mostly by strictly anaerobic microbes: Smithella, Ignavibacterium, Methanothrix, and Thiobacillus. Smithella includes the only known species—Smithella propionica, whose representatives are able to utilize propionic acid [66]. Ignavibacterium also includes the only one known species—Ignavibacterium album—a strictly anaerobic bacteria that uses carbohydrates, lipids, and proteins as a carbon source [67]. Methanothrix is an anaerobic archaea that performs methanogenesis. Thiobacillus are key functional microbes in fine chemical wastewater treatment systems [68]. Thiobacillus are autotrophic facultative anaerobic bacteria, known for their ability to couple denitrification to inorganic sulfur−compound oxidation [69]. Representatives of Thiobacillus are also able to utilize dimethyl sulfide, dimethyl disulfide, and carbon disulfide. Since the predicted functional gene profiles of the phytofilter zone had the highest abundance of functional genes responsible for denitrification, this area may play a major role in removing nitrogen compounds from the wastewater in the CW. 

The microbiome of the sedimentation tank differed strongly from those in other CW zones. It could be explained by the prevalence of anaerobic conditions and lack of vegetation in this area. The microbial community of the sedimentation tank was characterized by lower diversity indexes and higher relative abundances of Parcubacteria, Smithella, Proteiniclasticum, and Macellibacteroides. As in the previous zone, Smithella was also dominant in the microbiome of the sedimentation tank. Unlike other functional zones of the CW, sulfate reducers and sulfur bacteria belonging to Deltaproteobacteria phylum predominated in the microbial community of the sedimentation tank. Macellibacteroides are typical inhabitants of activated sludge in anaerobic tanks of typical municipal wastewater treatment plants [68]. Representatives of candidate phylum Parcubacteria (OD1) were detected in a wide range of natural environments, mainly with anaerobic conditions [70]. Parcubacteria are able to decompose cellulose and chitin, as well as to participate in hydrogen and sulfur cycles in anoxic sediments [71]. The sedimentation tank was characterized by higher relative abundances of genes responsible for methanogenesis, methanotrophy, and nitrification. In general, the predicted distribution of the functional genes related to methanogenesis and nitrification had a similar trend across the CW zones. It can be explained by the similarity of the methane monooxygenase (MMO) and ammonia monooxygenase (AMO) enzymes. Both enzymes are characterized by a similar kinetic turnover rate and an inhibition profile [71].

At the end of the CW, anaerobic Dechloromonas, Saccharibacteria genera, Clostridium, and Romboutsia were abundant within a sediment microbiome. Dechloromonas species are described as dissimilatory perchlorate reducing bacteria [72]. This genus includes four species oxidizing chlorobenzoate, toluene, xylene and capable to decompose benzene under anaerobic conditions [73]. Dechloromonas and Saccharibacteria are often found in activated sludge [68,74,75]; some representatives of Saccharibacteria phylum are able to decompose toluene [76]. Clostridium species are strictly anaerobic bacteria that can metabolize various compounds, such as carbohydrates, amino acids, alcohols, amino acids or purines [77]. Thus, microbial communities of different functional zones within a CW were completely different from the microbiomes (Caldilinea, Opitutus, and Prosthecobacter among dominant genera) of activated sludge obtained from wastewater treatment plants in the Moscow region [78].

Most of the potential human pathogens (Acinetobacter, Afipia, Arcobacter, Cloacibacillus, Escherichia, Mycobacterium, and Pseudomonas) were almost eliminated from wastewater when passing through the CW. In the outlet zone of the CW, Clostridium and Romboutsia were accumulated in the sediments. Both genera are Gram−positive microorganisms belonging to Clostridia class. Clostridia can form endospores which enable them to be dormant for extended periods under unfavorable conditions [79]. The pathogenicity of Romboutsia has not yet been confirmed [80], but many clostridia can be pathogenic to humans or animals [81]. Romboutsia are typical inhabitants of the human gastrointestinal tract [82]. We do not exclude contamination of the outlet zone of the CW by these two microorganisms from the lateral storm runoff. Nevertheless, the fact that these genera were not eliminated at the outlet of the CW indicates that more intensive wastewater treatments are required, such as: (1) longer retention time of the CW treatment, and (2) the introduction of additional treatment areas which would perform physical filtration and sedimentation of spores on organo−mineral particles.

## 5. Conclusions 

This study shows that despite the short retention time of wastewater in the given CW, functional zones with contrasting environmental and physicochemical conditions are characterized by distinct microbial composition and diversity. Among all the pollutants studied, only phthalates decomposed in the CW at such a short retention time of wastewater treatment. The rest of the pollutants were partially decomposed in different areas, but the main mechanisms for their removal from wastewater were sorption and sedimentation. The particle−size distribution analysis of the sediments revealed that specific self−organizing structures of root sludge are formed in phyto−treatment zones with the presence of vegetation, and these structures resemble the flocs of activated sludge. From the inlet to the outlet zones of the CW, a consistent increase in the specific surface area of particles was found.

The degree of spatial variability of the CW microbiome was controlled mainly by the movement of wastewater from the inlet to the outlet zones of the CW, as well as by the environmental conditions and vegetation cover that were performed for different functional zones of the CW. The functional zones with vegetation cover represented by Typha sp. and Phragmites sp. had the highest microbial diversity and were characterized by the highest number of microbial genera capable of decomposing organic pollutants. The microbial communities of phytofilers and sedimentation tank zones were represented mostly by strictly anaerobic microbes. The predicted functional gene profiles of the phytofilter zone had the highest abundance of functional genes responsible for denitrification, while the sedimentation tank was characterized by a higher predicted relative abundance of genes responsible for methanogenesis, methanotrophy, and nitrification. 

Unlike constructed wetlands, activated sludge systems have flow reactors with intensive mixing, which makes it possible to assess microbial activity by measuring metabolic products or balances of nutrients. In CWs, wastewater sampling in the intermediate area of functional zones is often impossible, so that it can be done only in inlet and outlet zones. Our study shows that molecular identification of microbiome can overсome these limitations of CWs and enables us to assess microbial processes in different functional zones of CWs. Thus, it can be used for industrial monitoring of constructed wetlands.

## Figures and Tables

**Table 1:** Characteristics of wastewaters entering the constructed wetland.

Characteristics of Wastewater	Range of Values during the Year
Temperature, °C	17.0–26.0
O_2_ (dissolved), mg L^−1^	2.1–8.3
pH	5.8–10.4
Electrical conductivity, µS cm^−1^	576–4201
NO_3_^−^, mg L^−1^	4.97–11.17
NO_2_^−^, mg L^−1^	0.095–0.345
NH_4_^+^, mg L^−1^	0.05–2.23
Total N , mg L^−1^	1.68–3.94
Fe^2+^, mg L^−1^	0.016–0.094
S^2−^, mg L^−1^	0.0013–0.0215
SO_4_^2−^, mg L^−1^	70.0–90.2
PO_4_^3−^, mg L^−1^	0.19–1.78
Cl^−^, mg L^−1^	48.5–50.7
Suspended substances (SS)mg L^−1^	9.0–180.0
Chemical oxygen demand (COD), mg L^−1^	10.6–83.8

**Table 2:** Functional zones of the constructed wetland.

Sample ID	Zone Index	Zone	Volume, m^3^	Sediment	Oxygen Conditions	Dominant Processes
I-1	I	Sand traps and grids	280	Washed sand	Aerobic	Mechanical retention, sedimentation of coarse suspended substances, organic matter (OM) oxidation;
I-2				Sand		
II-1	II	Phytofilters	2500	Silty sand	Aerobic	OM oxidation, nitrification;
II-2				Rooted sludge		
III-1	III	Sedimentation tank	3100	Bottom sludge	Anaerobic	Anaerobic oxidation of OM and sulfides, denitrification, anammox;
IV-1	IV	Phyto-treatment	580	Rooted sludge	Aerobic, locally anaerobic	All previous + biomass assimilation, nitrification;
IV-2				Sludge		
IV-3				Sludge		
V-1	V	Additional phyto-treatment	730	Rooted sludge	Aerobic, locally anaerobic	Biomass assimilation, OM oxidation, nitrification
V-2				Rooted sludge		

**Table 3:** Pearson’s correlation coefficients between the relative abundances of various microbial taxa at phylum, class, and genus levels and the physical characteristics of particles in the sediments (diameter and surface area of particles, the proportion of different size fractions). Abundant taxa with correlation coefficients > 0.5 or <−0.5 are indicated.

Taxon	Average Diameter	Surface Area	Diameter > 1000 µm	Diameter 100–1000 µm	Diameter 10–100 µm	Diameter 1–10 µm	Diameter < 1 µm
**Phyla**							
Acidobacteria	−0.41	0.46	−0.21	−0.37	0.33	0.41	0.49
Bacteroidetes	−0.45	0.36	−0.25	−0.47	0.49	0.34	0.13
Chloroflexi	−0.60	0.66	−0.30	−0.65	0.61	0.64	0.42
Firmicutes	−0.46	0.18	−0.64	−0.33	0.39	0.15	0.02
Proteobacteria	0.79	−0.50	0.74	0.71	−0.76	−0.47	−0.14
Verrucomicrobia	−0.40	0.51	−0.15	−0.40	0.34	0.49	0.49
Acidobacteria	−0.41	0.46	−0.21	−0.37	0.33	0.41	0.49
**Classes**							
Acidobacteria Gp17	−0.37	0.55	−0.14	−0.38	0.29	0.54	0.54
Acidobacteria Gp18	−0.27	0.44	0.00	−0.30	0.23	0.41	0.49
Acidobacteria Gp23	−0.31	0.65	−0.02	−0.37	0.24	0.64	0.76
Acidobacteria Gp3	−0.22	0.44	0.05	−0.20	0.11	0.39	0.65
Acidobacteria Gp6	−0.48	0.64	−0.23	−0.50	0.42	0.61	0.60
Acidobacteria Gp7	−0.33	0.60	0.00	−0.35	0.24	0.56	0.76
α−Proteobacteria	0.98	−0.75	0.87	0.93	−0.94	−0.75	−0.24
Anaerolineae	−0.58	0.66	−0.28	−0.63	0.58	0.63	0.44
Sphingobacteriia	−0.04	0.38	0.21	−0.02	−0.10	0.33	0.75
Verrucomicrobiae	−0.64	0.72	−0.42	−0.65	0.59	0.70	0.56
**Genera**							
Altererythrobacter	0.97	−0.70	0.91	0.90	−0.92	−0.70	−0.20
Cloacibacterium	0.71	−0.83	0.42	0.86	−0.81	−0.86	−0.36
Dechloromonas	−0.44	0.53	−0.34	−0.54	0.50	0.58	0.15
Flavobacterium	0.65	−0.54	0.60	0.75	−0.77	−0.58	0.05
Gp17	−0.37	0.55	−0.14	−0.38	0.29	0.54	0.54
Gp18	−0.27	0.44	0.00	−0.30	0.23	0.41	0.49
Gp6	−0.48	0.64	−0.23	−0.50	0.42	0.61	0.60
Gp7	−0.33	0.60	0.00	−0.35	0.24	0.56	0.76
Hydrogenophaga	0.99	−0.70	0.92	0.91	−0.94	−0.69	−0.19
Ignavibacterium	−0.44	0.55	−0.17	−0.50	0.45	0.55	0.38
Novosphingobium	0.97	−0.68	0.93	0.90	−0.94	−0.68	−0.14
Reyranella	−0.24	0.63	0.11	−0.30	0.16	0.59	0.89
Sphingobium	0.96	−0.84	0.79	0.98	−0.98	−0.84	−0.29
Sphingomonas	0.98	−0.68	0.92	0.89	−0.92	−0.67	−0.18
Sphingopyxis	0.99	−0.71	0.90	0.90	−0.93	−0.70	−0.22
Sphingorhabdus	0.98	−0.68	0.93	0.88	−0.92	−0.66	−0.18
Thiobacillus	−0.20	0.65	0.19	−0.31	0.15	0.64	0.82

## Supplementary Materials

The following are available online at https://www.mdpi.com/2076-2607/8/10/1604/s1, Table S1: Concentrations of individual PAH compounds in sediments of different functional zones across the constructed wetland. Table S2: Total extracted DNA concentrations and obtained number of nucleotide sequences in sediments of different functional zones across the constructed wetland.
