# Supplementary material for: Spatial Changes in Microbial Communities along Different Functional Zones of a Free-Water Surface Wetland
Source: Microorganisms. 2020 Oct 18;8(10):1604. doi: 10.3390/microorganisms8101604 (PMC7603099; doi:10.3390/microorganisms8101604)
Supplement: Supplementary file 1 [file microorganisms-08-01604-s001.pdf]

Supplementary Materials

Table S1. Concentrations of individual PAH compounds in sediments of different functional zones across the constructed wetland.

**PAHs concentration, µg g^{-1}**	**I-1**	**I-2**	**II-1**	**II-2**	**III-1**	**IV-1**	**IV-2**	**IV-3**	**V-1**
Fluorene + acenaphthene	0.084	4.486	1.837	0.992	2.179	1.682	2.093	1.624	1.524
Phenanthrene	0.057	2.518	1.823	1.329	1.666	1.259	1.593	1.589	1.621
Anthracene	0.008	0.198	0.658	0.414	0.581	0.534	0.436	0.560	0.568
Fluoranthene	0.027	0.846	1.722	1.277	1.783	1.403	1.785	1.574	1.531
Pyrene	0.014	0.960	1.127	0.652	0.867	0.695	0.769	0.823	0.850
Benz[a]anthracene	0.012	0.469	0.668	0.554	0.824	0.608	0.692	0.752	0.691
Chrysene	0.005	0.295	0.268	0.288	0.413	0.359	0.444	0.398	0.373
Benzo[b]fluoranthene	0.030	1.030	1.168	0.798	1.427	1.110	1.276	1.285	1.532
Benzo[k]fluoranthene	0.004	0.155	0.181	0.174	0.295	0.239	0.285	0.260	0.312
Benzo(a)pyrene	0.007	0.244	0.250	0.261	0.407	0.347	0.404	0.424	0.464

Table S2. Total extracted DNA concentrations and obtained number of nucleotide sequences in sediments of different functional zones across the constructed wetland.

**Functional zone**	**DNA concentration, ng µl^{-1}**	**Number of nucleotide sequences**
I-1	2.4	199427
I-2	24.8	173632
II-1	17.4	173994
II-2	38.3	79565
III-1	4.8	102192
IV-1	47.5	92408
IV-2	12.6	88831
IV-3	23.5	158823
V-1	11.8	80622
V-2	13.7	194288
